# Jejunojejunal Intussusception due to Metastatic Melanoma Seven Years after the Primer

**DOI:** 10.1155/2017/1237510

**Published:** 2017-09-12

**Authors:** Alexander Giakoustidis, Thomas Goulopoulos, Anastasios Boutis, George Kavvadias, Aristidis Kainantidis, Thomas Zaraboukas, Dimitrios Giakoustidis

**Affiliations:** ^1^Department of HPB Surgery, Royal London Hospital, London, UK; ^2^Department of Surgery, European Interbalkan Medical Centre, Thessaloniki, Greece; ^3^Department of Oncology, “Theagenion” Anti-Cancer Hospital, Thessaloniki, Greece; ^4^Department of Anesthesiology, European Interbalkan Medical Centre, Thessaloniki, Greece; ^5^Department of Pathology, European Interbalkan Medical Centre, Thessaloniki, Greece; ^6^Division of Transplant Surgery, Department of Surgery, Medical School, Aristotle University of Thessaloniki, Thessaloniki, Greece

## Abstract

Intestinal intussusception in adults is a rare medical condition accounting for less than 5% of all intussusceptions. Herein we present a 45-year-old patient with a history of abdominal pain and loss of weight. CT scan revealed jejunojejunal intussusceptions. The patient was subjected to exploratory operation and small intestine resection due to a mass causing intestinal intussusception. Pathology confirmed suspected diagnosis of metastatic melanoma to small intestine secondary to melanoma, 7 years after the initial manifestation. Postoperative evaluation with 18FDG-PET/CT revealed increased uptake in the thyroid gland. Subsequent total thyroidectomy revealed severe Hashimoto thyroiditis and no signs of metastasis. The patient received adjuvant immunotherapy and is healthy with no signs of recurrence 3 years after the initial diagnosis and treatment.

## 1. Introduction

Intussusception is a rare condition in the adult population accounting for less than 5% of all intussusceptions [1, 2]. Diagnosis is challenging because it often presents with nonspecific symptoms; abdominal pain with obstruction is the common presentation. Intussusception is responsible for 1% of all bowel obstructions [2, 3]. Surgery is the treatment of choice and in most of the cases there is underling malignant pathology involved [1–3]. Even though most small intestine tumors are usually metastatic, with metastatic melanoma being the most common, intussusceptions due to metastasis from melanoma have rarely been described in the literature. We herein present a case of jejunojejunal intussusceptions due to metastatic melanoma in a 45-year-old lady 7 years after the excision of the primer.

## 2. Case Presentation

**Figure 1:**
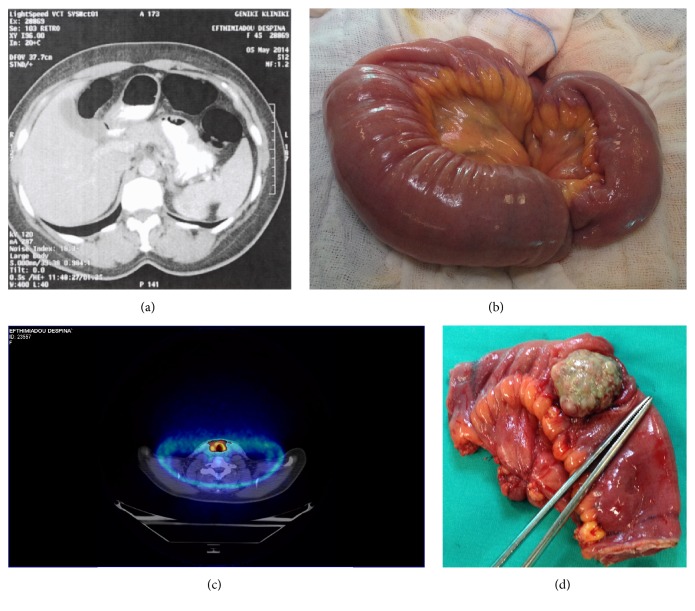
(a) CT scan showing an obstructing abdominal mass originating from the small bowel and causing intussusception; (b) intraoperative image of the intussusception; (c) thyroid gland with increased SUV in the 18FDG-PET/CT; (d) intestinal intussusceptions due to what appeared to be a distant melanoma metastasis.

A 45-year-old female presented to our clinic with abdominal pain for the last month. In addition the patient presented abdominal fullness, constipation, and loss of weight the last month. On physical examination abdominal discomfort was present. Medical history revealed a prior operation in the left arm for melanoma and axillary lymph node dissection 7 years ago with initial stage unknown and she received adjuvant high-dose interferon (Kirkwood regimen). In May 2014 she developed symptomatic iron-deficiency anemia and diffuse abdominal pain. Imaging exams revealed an obstructing abdominal mass originating from the small bowel and causing intussusception (Figure 1). Laboratory examination revealed no remarkable findings. An exploratory laparotomy was decided and performed. During the operation indeed an intestinal intussusception was found (Figure 1) due to what appeared to be a distant melanoma metastasis (Figure 1). Intestinal resection was performed with end-to-end hand sewn jejunojejunostomy. After careful checking no other distant metastasis was found in the abdomen. The patient had excellent postoperative course and was discharged at the 4th postoperative day.

**Figure 2:**
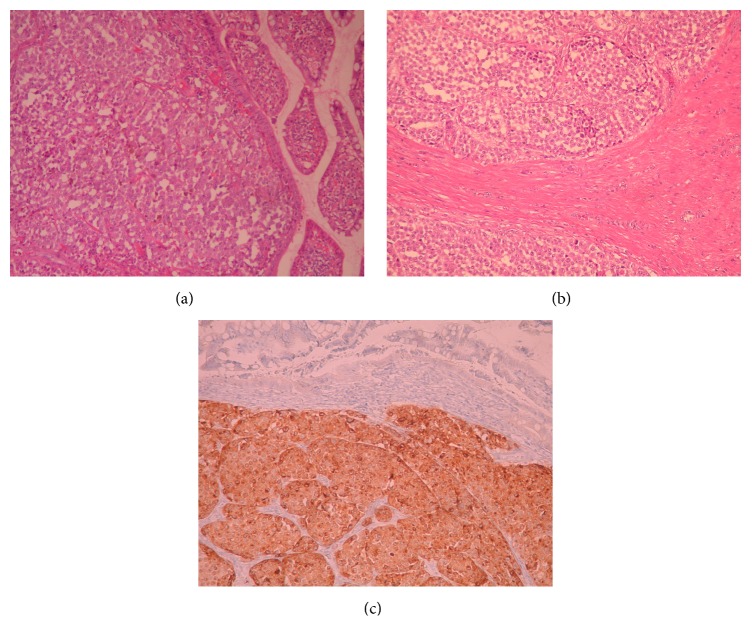
(a) Invasion of the mucosa of the small intestine by the melanoma cells (H+EX200). (b) Invasion of the muscularis propria by the melanoma cells (H+EX100). (c) Tumor cells are positive for HMB45 (immunostain ×200).

Histological examination of the tumor showed a malignant neoplasm consisting of large epithelioid atypical cells in solid arrangement which invades the mucosa, submucosa, and part of the muscularis propria. Immunohistochemistry revealed positivity of the tumor cells for HMB45 and Melan A (Figure 2). One month after the operation the patient was subjected to 18FDG-PET/CT in order to rule out any other distant metastases. 18FDG-PET/CT result was normal except the thyroid gland that showed increased uptake (Figure 1). Consequently it was decided to perform a total thyroidectomy. Two months after the first operation the patient was subjected to a total thyroidectomy. Pathology showed no signs of metastatic melanoma but revealed severe Hashimoto thyroiditis. The patient was referred for adjuvant immunotherapy. Molecular testing for the b-raf V600E mutation was positive. Between September and November 2014, she received first-line immunotherapy with ipilimumab at standard therapeutic regimen 3 mg/kg q3w for four cycles, which she tolerated without any remarkable toxicity. Subsequent radiologic and clinical controls showed no evidence of disease progression. The patient remains asymptomatic and free of disease 3 years after diagnosis and treatment of metastatic disease. The patient has given his consent for the case reports to be published.

## 3. Discussion

Intussusception in adults is a rare medical condition appearing in 5% of the total incidents of intussusceptions and represents the cause for 1% of intestinal obstructions [4]. The usual initial clinical signs are those of bowel obstruction while the diagnosis, in contrast with children, is difficult and in the majority of cases it is established intraoperatively [5]. Metastatic melanoma to the GI tract accounts for one-third of all abdominal metastases with small intestine being the most common site [4, 5] and is classified as stage four terminal diseases with an average lifespan of two months to 15 years following diagnosis [6]. Reported incidence of gastrointestinal metastases of melanoma in autopsy series reaches up to 60% [7]. On the contrary patients being clinically diagnosed with malignant melanoma and gastrointestinal metastases are limited to no more than 4% [8, 9]. The risk factors for malignant melanoma spread to the GI tract include superficial spreading melanoma, axial primary tumor, a Clark level III or IV, high degree of histologic regression, ulceration, and high mitotic rate [7, 10, 11]. Herein, we report an uncommon clinical presentation in adults, intussusception, with an underlying pathology, metastatic melanoma to the GI tract, that is rarely diagnosed premortem. This diagnosis should be considered in any patient who presents with gastrointestinal symptoms and a history of malignant melanoma. In our case a 45-year-old female presented with atypical symptoms of abdominal pain, constipation, and loss of weight. Previous history of melanoma in the left arm combined with the intestinal intussusceptions from the CT scan prompts us to operate on the patient with the increased suspicion of metastatic melanoma to the small intestine. Diagnosis was confirmed surgically and subsequently by pathology. The patient was restaged with 18FDG-PET/CT postoperatively showing increased uptake in the thyroid gland. This was proven false positive sign as we have already described, and this raises a concern over the evaluation of PET/CT. The patient completed the adjuvant immunotherapy and she is in an excellent condition 3 years after the initial diagnosis.
